# The Detrimental Effects of Peripartum Antibiotics on Gut Proliferation and Formula Feeding Injury in Neonatal Mice Are Alleviated with *Lactobacillus rhamnosus* GG

**DOI:** 10.3390/microorganisms11061482

**Published:** 2023-06-01

**Authors:** Alain Cuna, Marianne Nsumu, Heather L. Menden, Susana Chavez-Bueno, Venkatesh Sampath

**Affiliations:** 1 Division of Neonatology, Children’s Mercy Kansas City, Kansas City, MO 64108, USA; mnnsumu@cmh.edu (M.N.); hlmenden@cmh.edu (H.L.M.); vsampath@cmh.edu (V.S.); 2 School of Medicine, University of Missouri-Kansas City, Kansas City, MO 64108, USA; schavezbueno@cmh.edu; 3 Division of Infectious Diseases, Children’s Mercy Kansas City, Kansas City, MO 64108, USA

**Keywords:** necrotizing enterocolitis, antibiotics, probiotics, gut microbiome, postnatal gut adaptation

## Abstract

Peripartum antibiotics can negatively impact the developing gut microbiome and are associated with necrotizing enterocolitis (NEC). The mechanisms by which peripartum antibiotics increase the risk of NEC and strategies that can help mitigate this risk remain poorly understood. In this study, we determined mechanisms by which peripartum antibiotics increase neonatal gut injury and evaluated whether probiotics protect against gut injury potentiated by peripartum antibiotics. To accomplish this objective, we administered broad-spectrum antibiotics or sterile water to pregnant C57BL6 mice and induced neonatal gut injury to their pups with formula feeding. We found that pups exposed to antibiotics had reduced villus height, crypt depth, and intestinal olfactomedin 4 and proliferating cell nuclear antigen compared to the controls, indicating that peripartum antibiotics impaired intestinal proliferation. When formula feeding was used to induce NEC-like injury, more severe intestinal injury and apoptosis were observed in the pups exposed to antibiotics compared to the controls. Supplementation with the probiotic *Lactobacillus rhamnosus* GG (LGG) reduced the severity of formula-induced gut injury potentiated by antibiotics. Increased intestinal proliferating cell nuclear antigen and activation of the Gpr81-Wnt pathway were noted in the pups supplemented with LGG, suggesting partial restoration of intestinal proliferation by probiotics. We conclude that peripartum antibiotics potentiate neonatal gut injury by inhibiting intestinal proliferation. LGG supplementation decreases gut injury by activating the Gpr81-Wnt pathway and restoring intestinal proliferation impaired by peripartum antibiotics. Our results suggest that postnatal probiotics may be effective in mitigating the increased risk of NEC associated with peripartum antibiotic exposure in preterm infants.

## 1. Introduction

Antibiotics are among the most prescribed medications during pregnancy. It is estimated that ~40% of mothers receive antibiotics while pregnant [1] and that the incidence is increasing over time [2]. Common indications for maternal antibiotics include premature rupture of membranes [3], chorioamnionitis [4], prevention of maternal infections from cesarean or operative vaginal delivery [5], and prevention of early neonatal sepsis from group B *Streptococcus* [6]. Antibiotics are also prescribed to ~80% preterm infants shortly after birth, despite only a 2% incidence of culture-proven sepsis among very-low-birth-weight infants [7,8]. Widespread antibiotic exposure during the peripartum period can negatively impact early gut microbial colonization [9,10,11] and is associated with several childhood-onset diseases [12,13,14,15]. In preterm infants, the most widely recognized consequence of a perturbed gut microbiome is necrotizing enterocolitis (NEC) [16,17]. Several retrospective studies have demonstrated an association between peripartum antibiotics and an increased risk of NEC [18,19,20,21,22,23,24,25,26]. However, the mechanisms that explain this association remain poorly understood.

Probiotics are live, commensal bacteria that benefit the host when administered in enough quantities. Prophylactic supplementation of preterm infants with probiotics is increasingly being used to help decrease the risk of NEC [27,28,29,30,31,32]. Probiotics can suppress intestinal inflammation by inhibiting Toll-like receptor (TLR) 4 [33,34,35], decreasing inflammasome activity [36,37] and upregulating anti-inflammatory mediators such as interleukin (IL)-10 and TLR9 [38,39,40]. Probiotics can also augment intestinal barrier integrity by increasing mucin production [41,42] and enhancing tight junction protein function [43,44]. Other mechanisms of action include upregulation of TLR4 inhibitors such as SIGIRR [45,46,47], prevention of intestinal epithelial cell apoptosis [48], and colonization resistance against pathogenic bacteria [49]. Several clinical trials and mechanistic studies have demonstrated the beneficial effects of probiotics on the neonatal gut. However, whether probiotics remain effective in reducing NEC in the setting of peripartum antibiotics is poorly understood.

In this study, we investigated the impact of peripartum antibiotics and postnatal probiotics on NEC-like gut injury induced by formula feeding. We hypothesized that exposure to peripartum antibiotics increases gut injury, while postnatal probiotics can ameliorate gut injury potentiated by antibiotics. To test this hypothesis, we administered broad-spectrum antibiotics or sterile water to pregnant C57BL6 mice and subjected their newborn mouse pups to formula feeding to induce NEC-like injury. *Lactobacillus rhamnosus* GG (LGG), a probiotic commonly administered in preterm infants to help reduce NEC, was used for rescue experiments [50,51]. We also investigated potential mechanisms that mediate the impact of peripartum antibiotics and postnatal probiotics on NEC-like gut injury by assessing the gut mucosal barrier, permeability, inflammation, and epithelial proliferation.

## 2. Materials and Methods

Overview of animal experiments. We performed three sets of animal experiments: (1) a peripartum antibiotics experiment; (2) a peripartum antibiotics + formula feeding experiment; and (3) a peripartum antibiotics + probiotics + formula feeding experiment. All experiments were performed on C57BL/6 mice obtained from Charles River (Wilmington, MA, USA) and allowed to breed and deliver naturally. All animal experiments were performed according to local institutional committee guidelines with approval from the local IACUC (Protocol #1601-03).

**Figure 1:**
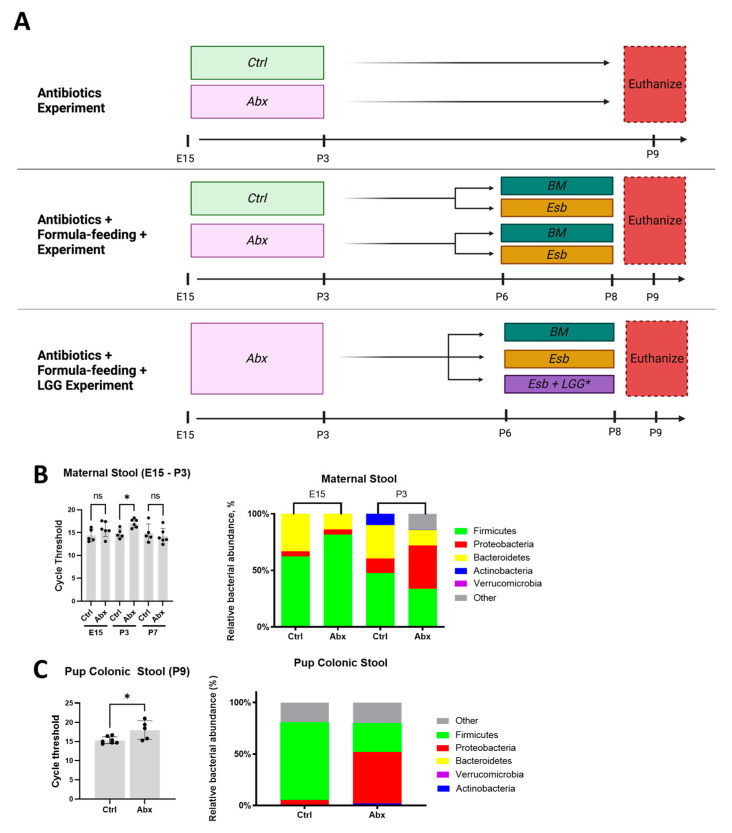
Overview of the experimental design and validation of the peripartum antibiotic protocol. (**A**) Schematic diagram of the three experiments performed in the study. Broad-spectrum antibiotics (Abx) or sterile water (Ctrl) were administered to pregnant dams via gavage from E15 to P3. BM = breastmilk; Esb = Esbilac; LGG = *Lactobacillus rhamnosus* GG. Note that in the antibiotic + formula feeding + LGG experiment, LGG was only administered to the Abx-Esb + LGG group by gavage from P4 to P8. All pups were sacrificed on P9. (**B**,**C**) Real-time qPCR for universal bacterial 16S rRNA and for major phyla was used to determine the relative gut bacterial load and abundance, respectively. (**B**) Stools from the dams were collected at E15 (baseline) and P3 (end of antibiotic treatment). (**C**) Colonic stools from the pups were collected at time of euthanasia at P9. The data presented as the means ± SD. * *p* < 0.05, using an unpaired Student’s *t*-test. ns, not significant. *n* = 5–7 stool samples for each group.

Peripartum antibiotics experiment. To determine the effect of peripartum antibiotics on postnatal gut development, we administered broad-spectrum antibiotics to pregnant dams by oral gavage once daily from embryonic day (E) 15 to postnatal day (P) 3 (Figure 1A). For the typical 21-day gestation in mice, this corresponds to treatment with antibiotics for 7 days or one-third of pregnancy plus an additional 3 days after delivery. The antibiotic cocktail consisted of ampicillin (5 mg/mL), neomycin (5 mg/mL), metronidazole (5 mg/mL), and vancomycin (2.5 mg/mL) as previously described [52]. Antibiotics were obtained from Sigma-Aldrich (St. Louis, MO, USA) and Gold Biotechnology (Olivette, MO, USA) and prepared fresh daily by reconstitution with sterile water. In this experiment, we compared pups of dams with antibiotic exposure (Abx group) to pups of dams administered sterile water (Ctrl group). 

Peripartum antibiotics + formula feeding experiment. To determine the impact of peripartum antibiotics on gut injury, we administered antibiotics or sterile water to pregnant dams from E15 to P3 as described above. We then administered formula feeding to the pups to induce NEC-like injury to the neonatal gut (Figure 1A). Our formula feeding protocol consisted of the pups being weaned from the dams at P6, housed in an incubator for temperature and humidity control, and gavage-fed with 0.12 mL of Esbilac (Esb, Pet-Ag, Inc., Hampshire, IL, USA) canine milk formula five times daily until P8 [46]. Littermate pups that remained with the dams and that were dam-fed with breastmilk (BM) served as controls. Together, these experiments resulted in four groups: the control + BM-fed group (Ctrl-BM), the control + Esb-fed group (Ctrl-Esb), the antibiotic + BM-fed group (Abx-BM), and the antibiotic + Esb-fed group (Abx-Esb).

Peripartum antibiotics + probiotics + formula feeding experiment. To determine whether probiotic supplementation protects against formula-induced gut injury among antibiotic-exposed pups, we first administered antibiotics to the pregnant dams from E15 to P3 as described above. We then administered the probiotic LGG at a dose of 0.1 mL of 10^8^ CFU/mL via oral gavage once daily from P4 to P8 [46]. LGG was obtained in freeze-dried form from American Type Culture Collection (ATCC#53103, Manassas, VA, USA) and grown as per the manufacturer’s recommendations. Lastly, the formula feeding protocol was initiated on P6 to P8 (Figure 1A). Pups from the same litter were used for comparison, resulting in three littermate-controlled groups: the antibiotic + BM-fed group (Abx-BM), the antibiotic + Esb-fed group (Abx-Esb), and the antibiotic + Esb-fed + LGG group (Abx-Esb + LGG). 

Sample collection and tissue processing. All pups were euthanized on P9, and distal ileum was collected for histology, qRT-PCR, Western blot, and immunofluorescence studies. Colonic tissue with stools from the pups was harvested and immediately frozen for further analysis. Stool samples from the dams were collected at E15, P3, and P7.

Gut microbiota composition analysis. The total genomic DNA from the maternal stools and mouse pup colonic stools were extracted using the QIAmp DNA stool kit (Qiagen, Valencia, CA, USA) according to the manufacturer’s instructions. Bacterial communities were detected by qPCR using 16s-rRNA-specific primers as described by Yang et al. [53]. A universal 16s rRNA primer was used to determine relative bacterial load; while phylum-specific primers for Firmicutes, Proteobacteria, Bacteroidetes, Actinobacteria, and Verrucomicrobia were used to determine relative bacterial abundance. 

Histological grading of tissue injury. Ileal tissue slides were stained with hematoxylin and eosin (H&E) and scanned into a computer using a Leica Biosystems Slide Scanner. A standardized 4-point scale was used to grade intestinal injury as previously described [45]. 

TUNEL assay. A terminal deoxynucleotidyl transferase dUTP nick end labeling (TUNEL) assay (Promega, Madison, WI, USA) was performed on terminal ileum slides as previously described [45]. The apoptotic index was calculated by dividing the total number of TUNEL-positive cells by the total number of 4′,6-diamidino-2-phenylindole (DAPI) stained cells. At least 2 slides per mouse and 3 to 5 fields per slide were analyzed.

qRT-PCR. Total RNA was extracted from the tissue samples using Invitrogen Tryzol Reagent (Fisher Scientific, Hampton, NH, USA) and cDNA prepared using a iScript cDNA synthesis kit (Bio-Rad, Hercules, CA, USA). Gene expression data were collected on a ViiA 7 Real-Time PCR (Applied Biosystems, Waltham, MA, USA) with SYBR Green Mastermix using pre-validated primers from MilliporeSigma. Relative gene expression was calculated for mucin 2 (MUC2), trefoil factor 3 (TFF3), epithelial cellular adhesion molecule (EPCAM), claudin 3 (CLDN3), olfactomedin 4 (OLFM4), proliferating cell nuclear antigen (PCNA), chemokine (C-X-C motif) ligand 1 (CXCL1), interleukin 6 (IL6), G protein-coupled receptor 81 (GPR81), and axin 2 (AXIN2) with the Pfaffl method [54]. The housekeeping genes ribosomal protein large, P0 (RPL0), and ß-actin were used for normalization.

Western Blot. Ileal samples were lysed in radioimmunoprecipitation assay (RIPA) buffer and then homogenized using a bullet blender. The antibodies used were as follows: mouse anti-ICAM-1 (Santa Cruz Biotechnology, Santa Cruz, CA, USA), rabbit anti-(p) p38 mitogen-activated protein kinases (MAPK), rabbit anti-p38 MAPK, rabbit anti-(p) p65, rabbit anti-p65 (Cell Signaling, Danvers, MA, USA), and mouse anti-ß-Actin (Sigma). ß-actin was used as a loading control. Blots were detected by chemiluminescence.

Immunofluorescence. Approximately 4 µm paraffin sections of intestine were deparaffinized in xylene and rehydrated with a graded series of alcohol. Antigen retrieval was performed using citrate buffer for 20 min at 95 °C. After several washes with PBS, the tissues were blocked with Power Block Universal Blocking Reagent for 1 h at room temperature. The tissue specimens were then stained overnight at 4 °C with primary rabbit anti-OLFM4, rabbit anti-PCNA, or rabbit anti-β-catenin. After being washed with PBS, the tissue sections were then incubated for 1 h at room temperature with secondary antibodies conjugated with Alexa fluor 568 or Alexa fluor 488 (ThermoFisher, Rockford, IL, USA). Images were acquired using a Zeiss Inverted LSM 510 meta laser scanning confocal microscope and image analysis was performed with ImageJ software (Version 1.53t).

In vivo intestinal permeability assay. Intestinal permeability was determined on the day of sacrifice at P9 by measuring the permeability of fluorescein isothiocyanate (FITC)-dextran in fasted animals as previously reported [55]. Briefly, the pups were administered 40–44 mg/100 g body weight suspended in sterile water by orogastric gavage. Four hours after, the pups were sacrificed and at least 100 µL of whole blood was collected in an Eppendorf tube. The blood was centrifuged at 8000 rpm (10,000× *g*) for 10 min. Serum was collected and diluted 1:4 in water. To each well, 100 µL of the diluted serum or prepared standard curve samples was added. Fluorescence was measured in a plate reader with 485 excitation/528 emission, and permeability values were calculated based on the standard curve. 

Data analysis. Data are presented as the means ± SD or the median with the interquartile range. All animal data were obtained with 6–13 animals for each experimental group. RNA quantification and the PCR results were performed in triplicate. Statistical analysis was carried out using GraphPad Prism (San Diego, CA, USA) with statistical significance set at *p* < 0.05. The Kruskal–Wallis test, analysis of variance, or two-tailed Student’s *t*-tests were performed, with correction for multiple testing as appropriate.

## 3. Results

### 3.1. Administration of Peripartum Antibiotics Results in Maternal and Neonatal Gut Dysbiosis

We first evaluated whether our model of peripartum antibiotic administration was sufficient to alter the intestinal microbiome of dams and their newborn pups. Real-time qPCR for bacterial 16S rRNA primers was used to determine the relative gut bacterial load and abundance. Prior to starting antibiotics at E15 (baseline), the gut bacterial load was similar between the Ctrl and Abx dams. By the end of peripartum antibiotic treatment at P3, however, higher cycle threshold values for detecting bacterial 16s rRNA, which indicates reduced gut bacterial load, were noted in Abx dams compared to Ctrl dams (Figure 1B). Antibiotic exposure also altered gut bacterial composition at the phylum level, resulting in increased Proteobacteria and reduced Bacteroidetes in the Abx dams compared to the Ctrl dams (Figure 1B). In the pups, the colonic stools obtained immediately following euthanasia on P9 revealed a similar pattern of reduced gut bacterial load in the Abx group compared to the Ctrl (Figure 1C). In addition, peripartum antibiotics resulted in increased Proteobacteria and decreased Firmicutes in the pups (Figure 1C). Together, these data show that our antibiotic regimen caused significant alteration in the gut microbiota of the dams and pups characterized by reduced bacterial load and dysbiotic enrichment of potential pathobionts.

### 3.2. Peripartum Antibiotics Impair Intestinal Proliferation of the Developing Gut without Affecting the Gut Mucosal Barrier or Gut Permeability

**Figure 2:**
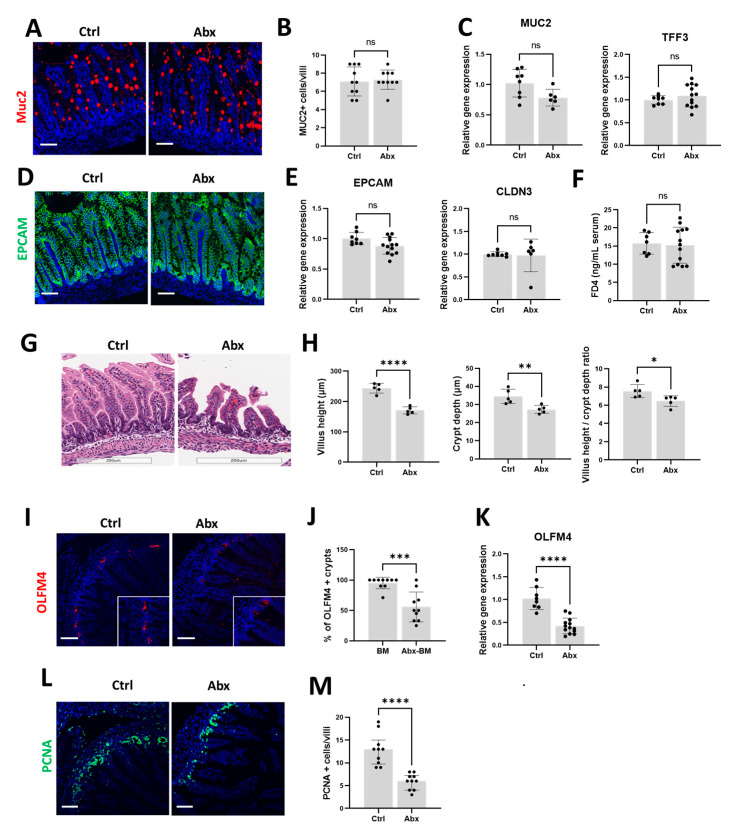
Effects of peripartum antibiotics on the developing neonatal gut of the mouse pups under baseline conditions. Broad-spectrum antibiotics or sterile water were given to pregnant dams from E15 to P3. Ileal tissue was harvested from the pups at P9. Pups exposed to antibiotics (Abx) were compared to unexposed pups (Ctrl). (**A**–**C**) Effects on intestinal mucin. (**A**) Representative micrograph of immunofluorescence staining of terminal ileum for MUC2 (red). (**B**) Quantification of MUC2+ cells/villi. (**C**) Relative gene expression of MUC2 and TFF3. (**D**–**F**) Effects on intestinal tight junction proteins and intestinal permeability. (**D**) Representative micrograph of immunofluorescence staining of terminal ileum for EPCAM (green). (**E**) Relative gene expression of EPCAM and CLDN3. (**F**) Measurement of fluorescein isothiocyanate-dextran 4 kDa (FD4) levels in serum 4 h after oral gavage. (**G**–**M**) Effects on intestinal proliferation. (**G**) Representative images of terminal ileum stained with H&E. (**H**) Measurements of villus height, crypt depth, and villus height to crypt depth ratio obtained from five well-oriented villi and crypts of the terminal ileum. (**I**,**J**) Immunofluorescence staining and quantification for OLFM4 (red) in the terminal ileum. (**K**) Reduced gene expression of OLFM4 in the terminal ileum of the Abx group. (**L**,**M**) Immunofluorescence staining and quantification for PCNA (green) in the terminal ileum. The data are presented as the means ± SD. * *p* < 0.05; ** *p* < 0.01; *** *p* < 0.001; **** *p* < 0.0001, using an unpaired Student’s *t*-test. ns, not significant. *n* = 8–13 pups for each group. Scale bar for all images is 50 µm.

We then investigated the effects of peripartum antibiotics on the developing neonatal gut under baseline conditions, focusing specifically on the gut mucosal barrier, gut permeability, and intestinal proliferation. We found that the expression of MUC2 and TFF3, key components of the gut mucosal barrier, was similar between the Ctrl and Abx groups (Figure 2A–C). We also found that gut permeability, as evaluated by immunofluorescence staining and gene expression analysis of EPCAM and CLDN3 (Figure 2D,E) and by the intestinal permeability assay with FITC-dextran (Figure 2F), was similar between the Ctrl and Abx groups. In contrast, we observed a reduction in villus height, crypt depth, and the villus height to crypt depth ratio in the Abx group compared to the Ctrl group (Figure 2G,H). As impaired maintenance of villus height and crypt depth could result from reduced intestinal proliferation, we investigated the impact of antibiotic exposure on olfactomedin 4 (OLFM4), a surrogate marker of intestinal stem cell population [56]. We found that the Abx group had a decreased percentage of crypts expressing OLFM4 (Figure 2I,J) as well as reduced total OLFM4 gene expression (Figure 2K) compared to the Ctrl group. Immunofluorescence staining of the terminal ileum for proliferating cell nuclear antigen (PCNA), a marker of cellular proliferation, was also decreased with antibiotic exposure (Figure 2L,M). Collectively, these results indicate that exposure to peripartum antibiotics impaired villus development related to diminished intestinal epithelial cell proliferation, while the gut mucosal barrier and gut permeability remained unchanged. 

### 3.3. Peripartum Antibiotics Potentiate Neonatal Gut Injury from Formula Feeding

**Figure 3:**
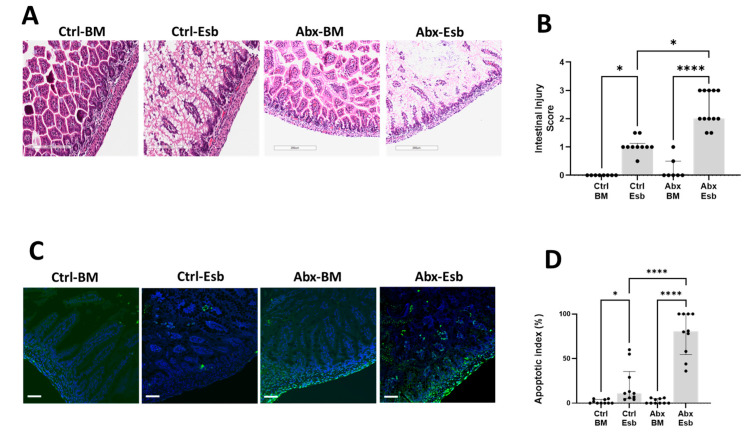
Detrimental effects of peripartum antibiotics on neonatal gut injury in the mouse pups subjected to formula feeding. The antibiotic-exposed pups were formula-fed with Esbilac from P6 to P8. Terminal ileum samples were obtained after sacrifice on P9. (**A**) Representative images of H&E-stained terminal ileum sections. (**B**) Intestinal injury scores showing more severe injury in the Abx-Esb group compared to the Ctrl-Esb group. (**C**) Representative images of TUNEL-stained terminal ileum sections. TUNEL-positive cells indicative of apoptosis are indicated by green fluorescence. (**D**) Apoptotic index showing worse intestinal apoptosis in the Abx-Esb group compared to the Ctrl-Esb group. The data are presented as the median and interquartile ranges. * *p* < 0.05; **** *p* < 0.0001, using the Kruskal–Wallis test and with correction for multiple comparisons. *n* = 7–12 pups per experimental group. Scale bar is 200 µm for histology and 50 µm for immunofluorescence.

Positing that antibiotic exposure will enhance vulnerability to intestinal injury in neonatal pups, we challenged the antibiotic-exposed and unexposed neonatal pups to formula feeding. We found that while formula feeding with Esbilac induced mild intestinal injury in the Ctrl-Esb group, the same formula feeding protocol resulted in more severe intestinal injury in the Abx-Esb group (Figure 3A,B). A similar pattern of worse apoptotic injury in the Abx-Esb group was also found compared to the Ctrl-Esb group (Figure 3C,D). Together, these findings indicate that peripartum antibiotics exposure worsened NEC-like injury induced by formula feeding in the developing neonatal gut. 

### 3.4. Worse Gut Injury with Peripartum Antibiotics Is Not Associated with Increased Inflammation Nor with Reduced Mucin or Tight Junction Protein Expression

**Figure 4:**
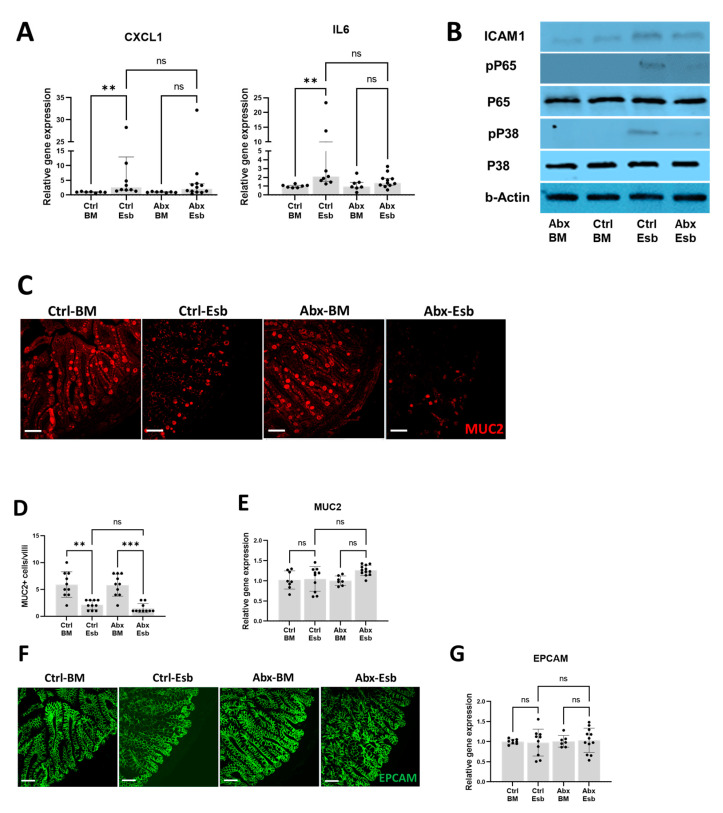
Effects of peripartum antibiotics on intestinal inflammation, intestinal mucin, and intestinal tight junction proteins following formula feeding injury. (**A**,**B**) Effects on intestinal inflammation. (**A**) Intestinal gene expression of CXCL1 and IL6 (inflammatory markers). Note higher markers for intestinal inflammation in the Ctrl-Esb group but not in the Abx-Esb group. (**B**) Western blot for ICAM1 (inflammatory marker) and p38 and p65 (TLR4 signaling markers). Note increased ICAM1, p38, and p65 protein expression in the Ctrl-Esb group but not in the Abx-Esb group. (**C**–**E**) Effects on intestinal mucin. (**C**) Representative micrographs of immunofluorescence staining for MUC2 (red) in the terminal ileum. (**D**) Quantification of MUC2+ cells/villi. (**E**) Relative gene expression of MUC2. (**F**,**G**) Effects on intestinal tight junction proteins. (**F**) Representative micrographs of immunofluorescence staining for ECPAM (green) in the terminal ileum. (**G**) Relative gene expression of EPCAM. The data are presented as the mean ± SD or the median and interquartile range. ** *p* < 0.01; *** *p* < 0.001, using one-way ANOVA or the Kruskal–Wallis test as appropriate and with correction for multiple comparisons. ns, not significant. *n* = 7–12 pups per experimental group. Scale bar is 50 µm for all images.

To investigate the potential mechanisms underlying the detrimental effect of peripartum antibiotics on NEC-like gut injury, we first evaluated whether TLR-mediated intestinal inflammation was increased in the Abx-Esb group. The rationale for this evaluation is because exaggerated TLR-mediated inflammation is a major mechanism of injury in NEC [57]. Interestingly, we found that while formula feeding increased intestinal expression of CXCL1 and IL6 in the Ctrl-Esb group, the same pro-inflammatory markers were not significantly increased in the Abx-Esb group (Figure 4A). We also found less TLR4-mediated inflammation in the Abx-Esb group compared to the Ctrl-Esb group, as evaluated by Western blot analysis for TLR4 signaling markers (phosphorylated p38 and phosphorylated p65) and for the inflammatory marker intercellular adhesion molecule-1 (ICAM-1) (Figure 4B). These results suggest that intestinal inflammation was not the primary mechanism by which peripartum antibiotics worsened NEC-like injury. 

We continued our investigation of potential mechanisms by which antibiotics worsen NEC-like gut injury by evaluating MUC2 and EPCAM. We found that formula feeding reduced MUC2 expression in the terminal ileum (Figure 4C); however, the degree of reduction in MUC2 was similar between the antibiotic-exposed pups and the non-exposed pups (Figure 4D). Overall intestinal gene expression of MUC2 also remained similar among the different groups (Figure 4E). We also found that intestinal EPCAM expression was similar regardless of formula feeding and antibiotic interventions in both immunofluorescence studies (Figure 4F) and gene expression analysis (Figure 4G). These results suggest that worse gut injury with peripartum antibiotics could not be explained by impairments in gut mucin or tight junction protein expression.

### 3.5. Peripartum Antibiotics Potentiate NEC-like Injury by Causing Impairments in Intestinal Proliferation

**Figure 5:**
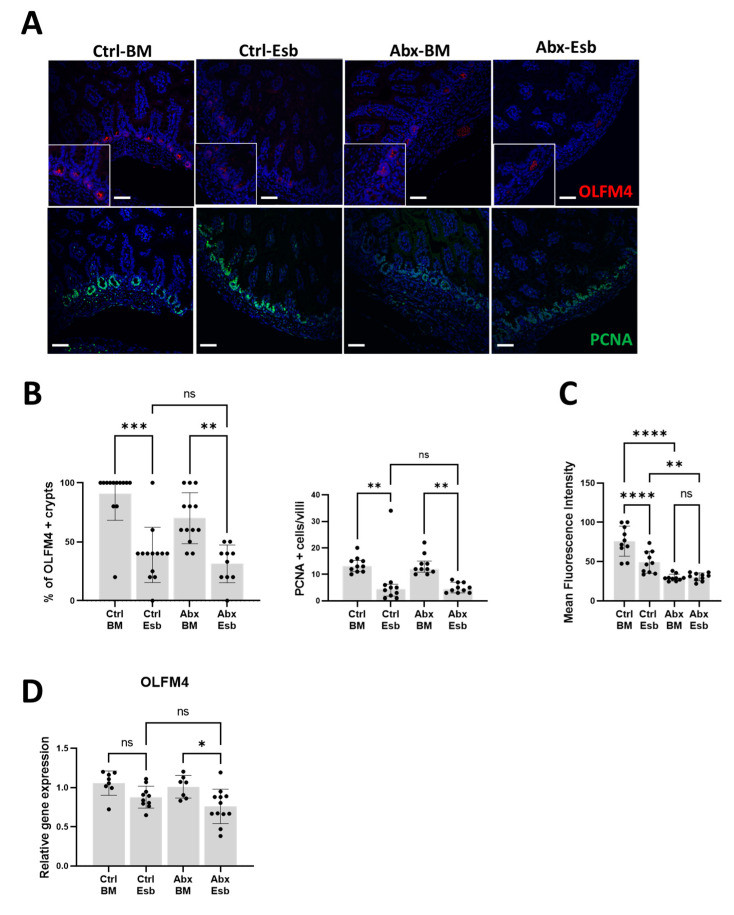
Detrimental effects of peripartum antibiotics and formula feeding on intestinal proliferation. (**A**) Representative micrographs of immunofluorescence staining for OLFM4 (red) and PCNA (green) in the terminal ileum. (**B**) Quantification of OLFM4+ crypts and PCNA+ cells/villi. (**C**) Quantification of the mean fluorescence intensity of PCNA by ImageJ software. (**D**) Gene expression data demonstrating a reduction in intestinal OLFM4 (marker of intestinal stem cell population) in the Abx-Esb group. The data are presented as the mean ± SD or the median and interquartile range. * *p* < 0.05; ** *p* < 0.01; *** *p* < 0.001; **** *p* < 0.0001, using one-way ANOVA or the Kruskal–Wallis test as appropriate and with correction for multiple comparisons. *n* = 7–12 pups per experimental group. Scale bar is 50 µm for all images.

Informed by our earlier results, we hypothesized that impaired intestinal proliferation is the potential mechanism by which peripartum antibiotics potentiate NEC-like injury from formula feeding. To test this hypothesis, we evaluated the impact of antibiotics on markers of intestinal proliferation in the setting of formula feeding injury. Using immunofluorescence staining, we found a decrease in the number of crypts positive for OLFM4 and PCNA with formula feeding (Figure 5A,B). While the degree of reduction in the OLFM4+ and PCNA+ cells was comparable between the Abx-Esb and Ctrl-Esb groups, we observed that the fluorescence signal intensity for PCNA [58] was noticeably decreased with antibiotic exposure (Figure 5C). Gene expression analysis also demonstrated a significant reduction in intestinal OLFM4 among the pups exposed to both antibiotics and formula feeding (Figure 5D). Taken together, these results suggest that while formula feeding by itself reduces intestinal proliferation, exposure to peripartum antibiotics impairs intestinal proliferation even further, contributing to the severe NEC-like injury observed in the Abx-Esb pups. 

### 3.6. The Probiotic LGG Decreases NEC-like Injury Potentiated by Peripartum Antibiotics through Activation of the Gpr81-Wnt-β-Catenin Pathway

**Figure 6:**
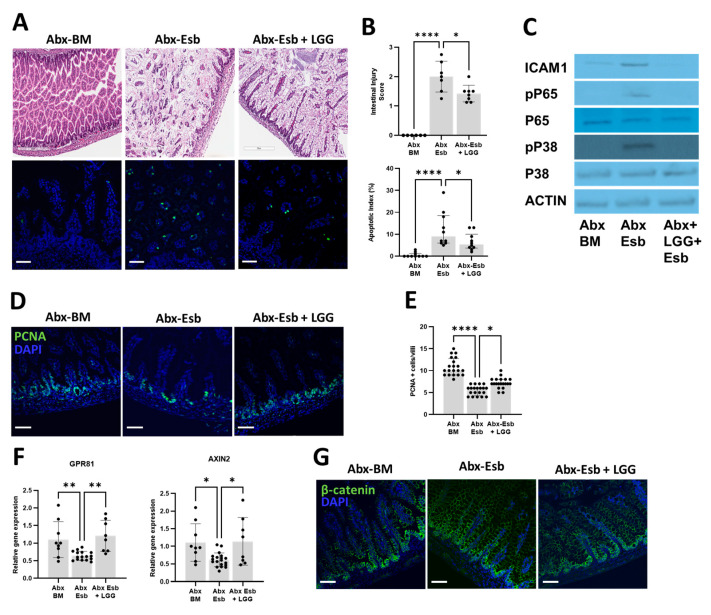
Beneficial effects of postnatal LGG on formula feeding injury potentiated by peripartum antibiotics. LGG was administered by gavage from P4 to P8 to antibiotic-exposed pups subjected to formula feeding. (**A**–**C**) Effects of LGG on intestinal injury. (**A**) Representative H&E-stained and TUNEL-stained images of the terminal ileum. (**B**) Intestinal injury scores and apoptotic indices. (**C**) Representative Western blot images for the protein expression of ICAM1, p65, and p38. (**D**,**E**) Effects of LGG on intestinal proliferation. (**D**) Representative micrograph of immunofluorescence staining for PCNA (green) in the terminal ileum. (**E**) Quantification of PCNA+ cells/villi. (**F**,**G**) Activation of the Gpr81-Wnt-β-catenin pathway by LGG. (**F**) Intestinal gene expression of GPR81 (a receptor for lactate) and AXIN2 (a marker for b-catenin). (**G**) Representative micrograph of immunofluorescence staining for b-catenin (green). Note how the pattern of prominent β-catenin expression in the crypt region of the Abx-BM group is diminished in the Abx-Esb group and partially restored in the Abx-Esb + LGG group. The data are presented as the mean ± SD or the median and interquartile range. * *p* < 0.05; ** *p* < 0.01; **** *p* < 0.0001, using one-way ANOVA or the Kruskal–Wallis test as appropriate and with correction for multiple comparisons. *n* = 6–8 pups per experimental group. Scale bar is 200 µm for histology and 50 µm for immunofluorescence.

Lastly, we asked whether postnatal supplementation with LGG would be effective in reducing NEC-like injury potentiated by peripartum antibiotics. To answer this question, we used littermate pups exposed to peripartum antibiotics and divided them into three groups: the BM-fed group (Abx-BM), the Esb-fed group (Abx-Esb), and the Esb-fed + LGG group (Abx-Esb + LGG). We found that the severity of intestinal injury, apoptosis, and TLR4-mediated inflammation from antibiotics + formula feeding were reduced with LGG supplementation (Figure 6A–C). We then investigated the possible mechanism by which LGG decreased gut injury from antibiotics + formula feeding. Based on our results demonstrating the detrimental effects of peripartum antibiotics on intestinal proliferation, we hypothesized that LGG decreases NEC-like injury via stimulation of intestinal proliferation. To test this hypothesis, we again evaluated intestinal proliferation with immunofluorescence staining for PCNA. We found that LGG supplementation increased PCNA expression in the terminal ileum of the Abx-Esb + LGG group compared to the Abx-Esb group (Figure 6D,E). Taken together, these results suggest that postnatal LGG ameliorates NEC-like injury by restoring intestinal proliferation impaired by peripartum antibiotics.

Previous studies have demonstrated that lactic-acid-producing bacteria such as LGG activate the G-protein-coupled receptor 81 (Gpr81) to stimulate intestinal proliferation through the Wnt/β-catenin pathway [59]. We thus investigated whether LGG supplementation in our model increased intestinal proliferation through the Gpr81-Wnt/β-catenin pathway. We evaluated the gene expression of GPR81 and AXIN2 (a marker of β-catenin activity) by qPCR. We found that formula feeding significantly reduced the gene expression of GPR81 and AXIN2, while supplementation with LGG increased the gene expression levels of GPR81 and AXIN2. (Figure 6F). We also assessed β-catenin activity by immunofluorescence staining of the terminal ileum. We found that formula feeding reduced the expression of β-catenin in the intestinal crypts in the Abx-Esb group, while supplementation with LGG partially restored the normal pattern of preferential crypt expression of β-catenin in the Abx-Esb + LGG group (Figure 6G). Taken together, these experiments suggest that the mechanism by which LGG increases intestinal proliferation is by activation of the lactate-Gpr81-Wnt pathway.

## 4. Discussion

This study investigated the effects of peripartum antibiotics and postnatal probiotics on neonatal mice subjected to formula feeding to induce NEC-like gut injury. We found that peripartum antibiotics worsened gut injury from formula feeding, while the probiotic LGG reduced the severity of gut injury potentiated by peripartum antibiotics. We also found that peripartum antibiotics impaired intestinal proliferation, while postnatal LGG partially restored intestinal proliferation impaired by peripartum antibiotics in conjunction with activation of the Gpr81-Wnt-ß-catenin pathway. Taken together, our findings provide mechanistic insights underlying the increased risk of NEC observed in preterm infants exposed to peripartum antibiotics. Our findings also provide supporting evidence that probiotics remain beneficial in reducing NEC in the setting of peripartum antibiotics exposure.

Other studies have also used mouse models to demonstrate the harms of peripartum antibiotics on the neonatal gut. Chen et al. [60] used a model of maternal antibiotic treatment during pregnancy, starting from E15 to the time of delivery, and identified several impairments in the intestinal development of their pups, including increased intestinal inflammation, a reduced number of Goblet cells and PCNA cells, and reduced expression of tight junction proteins. Chaaban et al. [61] used a different model—that of postnatal antibiotic treatment directly administered to newborn pups via intraperitoneal injections starting from P1 to P10—and likewise detected several intestinal impairments, including reduced villus height, impaired intestinal proliferation, and a reduced number of goblet cells and Paneth cells. Chaaban et al. also conducted NEC experiments by oral bacterial challenge with *Klebsiella pneumonia* and found worse intestinal injury with antibiotics compared to controls. In our study, we administered antibiotics to pregnant dams from E15 to P3 to mimic peripartum antibiotic exposure in preterm infants, which often starts prenatally and extends postnatally for 48–72 h, while awaiting the results of blood cultures. We also induced NEC-like injury but used formula feeding instead of oral bacterial challenge. Despite the differences in experimental design, comparable results of more severe intestinal injury were found in our study, thus lending further credence that peripartum antibiotics have detrimental effects on the developing gut which increase the risk of developing NEC.

Previous studies that have investigated probiotics in the setting of peripartum antibiotic exposure have mostly focused on their effects on the neonatal gut microbiota [62,63]. While these studies indicate that probiotic supplementation can favorably alter the composition of the gut microbiome disrupted by antibiotics, studies showing direct beneficial effects on the neonatal gut are lacking. In our study, we tested the effects of supplementation with LGG as a rescue intervention to alleviate the harmful effects of peripartum antibiotics on the neonatal gut. We chose LGG because (1) *Lactobacillus* species are the predominant colonizers of the murine gut immediately after birth [46,64,65]; (2) probiotics including LGG are increasingly being used in preterm infants to prevent NEC [27,28,31]; and (3) lactate-producing bacteria such as LGG have been shown in other studies to promote gut proliferation [59]. We found that supplementation with LGG partially restored intestinal proliferation impaired by antibiotics and reduced gut injury from formula feeding. Thus, in addition to favorable alteration of the gut microbiota identified by previous studies, our study provides evidence that probiotic supplementation after peripartum antibiotics also has direct beneficial effects on neonatal intestinal tract physiology.

In investigating the mechanisms of how peripartum antibiotics potentiated NEC-like injury from formula feeding, a consistent theme of impaired intestinal proliferation induced by peripartum antibiotics emerged. These findings are consistent with recent studies that used germ-free and/or antibiotic-treated mice to demonstrate how disruption of early life microbiota negatively impacts intestinal proliferation [66,67]. Which specific microbial communities drive the proper establishment of intestinal proliferation in the neonatal gut remains unknown. In our study, Firmicutes were significantly decreased with peripartum antibiotics, suggesting that bacterial communities from this phylum could be important mediators of intestinal proliferation. Interestingly, *Lactobacillus* species such as LGG belong to the phylum Firmicutes, and LGG supplementation increases intestinal proliferation in conjunction with activation of Wnt signaling—a known regulator of intestinal proliferation [68,69]. Thus, in interpreting the main findings of our study, we hypothesize that peripartum antibiotics prevented colonization of the neonatal gut with commensal bacteria such as *Lactobacillus*, which produce lactate and other microbial metabolites important for postnatal establishment of intestinal proliferation [59]. The resulting impairment of intestinal proliferation hampered the capability of the neonatal gut for regeneration, thus causing increased gut injury and apoptosis with formula feeding. Supplementation with LGG re-established lactate-producing bacteria normally abundant in the neonatal gut, restored intestinal proliferation via the lactate-Gpr81-Wnt pathway, and ameliorated gut injury and apoptosis potentiated by peripartum antibiotics.

We also explored other mechanisms that may explain the worse injury with peripartum antibiotics. We found that intestinal TLR-mediated inflammation was decreased with antibiotics, suggesting that inflammation was not the primary mechanism driving the worse gut injury observed in the antibiotic-exposed pups. The decreased gut inflammation noted in our study was likely because of the antibiotics decreasing the bacterial load which activates TLR in the gut. We also found that expression of intestinal mucin and tight junction proteins, and gut permeability assessed by FITC-dextran, were not significantly altered with peripartum antibiotics. Taken together, these results suggest that neither exaggerated intestinal inflammation nor a diminished gut mucosal barrier and gut permeability were primary contributors to the worse injury with peripartum antibiotics in our model. Our results indicate that exposure to noxious stimuli (such as formula feeding) in the setting of impaired intestinal proliferation could also result in NEC-like injury. These results differ from studies which identified a defective gut mucosal barrier, impaired gut permeability, and uncontrolled intestinal inflammation as central players in NEC development [70,71]. Our findings thus suggest that impaired intestinal proliferation is another potential pathway by which NEC can occur. Our findings also differed from other studies which observed a reduction in Goblet cells and tight junction proteins with peripartum antibiotics [60,61]. We speculate that the differences in our model, such as the duration of antibiotic treatment (limited to postnatal day 3), the route of administration (oral vs. intravenous), and/or our use of broad-spectrum antibiotics (ampicillin, vancomycin, neomycin, and metronidazole), could have contributed to the differences in these results.

We acknowledge several limitations in our study. One limitation was the use of qPCR for specific 16S bacterial RNA targets [53], which allowed us to demonstrate only major changes in gut microbiota composition at the phylum level. We opted for this approach because our main objective was to determine the functional effects of peripartum antibiotics and probiotics supplementation on neonatal gut function. A second limitation was our use of antibiotics which are broader and of longer duration than typical peripartum antibiotics administered to mothers and preterm infants. Whether our findings could be translated to what is seen clinically remains undetermined. Additional studies to assess the impact of limited duration, narrow-spectrum antibiotics on the neonatal gut’s microbiome and vulnerability to NEC are underway to better address this limitation. Another limitation was that we focused our investigation on the effects of LGG on intestinal proliferation. We acknowledge that other probiotics besides LGG can have beneficial effects post antibiotics and that other mechanisms besides enhanced intestinal proliferation can mediate these benefits.

## 5. Conclusions

In summary, we have shown that peripartum antibiotics can impair intestinal proliferation and negatively affect the neonatal gut’s ability to regenerate following injury from formula feeding. We have also shown that postnatal supplementation with the probiotic LGG can help ameliorate the negative impact of antibiotics on intestinal proliferation and reduce gut injury. Our findings in mice support accumulating evidence from human studies regarding the increased risk of NEC with peripartum antibiotic exposure [18,22,26,72]. Our findings also provide supporting evidence that supplementation with probiotics may be an effective strategy to help offset the harmful effects of antibiotics.

Future directions of research in this area include studies to identify which specific gut microbiota and/or gut microbial metabolites are altered by peripartum antibiotics and what their role is in the postnatal establishment of intestinal proliferation. Knowledge gained from these future studies may help guide the development of microbial-based strategies that ameliorate disruptions caused by antibiotics and other stressors during postnatal gut adaptation.

## Data Availability

The data presented in this study are available from the corresponding author upon request.
